# K‐mer counting and curated libraries drive efficient annotation of repeats in plant genomes

**DOI:** 10.1002/tpg2.20143

**Published:** 2021-09-25

**Authors:** Bruno Contreras‐Moreira, Carla V Filippi, Guy Naamati, Carlos García Girón, James E Allen, Paul Flicek

**Affiliations:** ^1^ European Molecular Biology Laboratory, European Bioinformatics Institute Wellcome Genome Campus Hinxton Cambridge CB10 1SD UK; ^2^ Instituto de Biotecnología, Centro de Investigaciones en Ciencias Veterinarias y Agronómicas (CICVyA), Instituto Nacional de Tecnología Agropecuaria (INTA); Instituto de Agrobiotecnología y Biología Molecular (IABIMO) INTA‐Consejo Nacional de Investigaciones Científicas y Técnicas (CONICET) Nicolas Repetto y Los Reseros s/n (1686) Hurlingham Buenos Aires Argentina; ^3^ CONICET Av Rivadavia 1917, C1033AAJ Ciudad de Buenos Aires Argentina

## Abstract

The annotation of repetitive sequences within plant genomes can help in the interpretation of observed phenotypes. Moreover, repeat masking is required for tasks such as whole‐genome alignment, promoter analysis, or pangenome exploration. Although homology‐based annotation methods are computationally expensive, k‐mer strategies for masking are orders of magnitude faster. Here, we benchmarked a two‐step approach, where repeats were first called by k‐mer counting and then annotated by comparison to curated libraries. This hybrid protocol was tested on 20 plant genomes from Ensembl, with the k‐mer‐based Repeat Detector (Red) and two repeat libraries (REdat, last updated in 2013, and nrTEplants, curated for this work). Custom libraries produced by RepeatModeler were also tested. We obtained repeated genome fractions that matched those reported in the literature but with shorter repeated elements than those produced directly by sequence homology. Inspection of the masked regions that overlapped genes revealed no preference for specific protein domains. Most Red‐masked sequences could be successfully classified by sequence similarity, with the complete protocol taking less than 2 h on a desktop Linux box. A guide to curating your own repeat libraries and the scripts for masking and annotating plant genomes can be obtained at https://github.com/Ensembl/plant‐scripts.

## Core Ideas

- Control Pfam domains minimize unrelated coding sequences in repeat libraries.
- Repeat calling by k‐mer counting with Red does not preferentially mask NLR genes.
- Repeats called by Red can be efficiently classified by sequence similarity with minimap2.

**Abbreviations**
NLR: nucleotide‐binding and leucine‐rich repeat immune receptor
R genes: disease resistance genes
Red: Repeat Detector
RepMod: RepeatModeler
RM: RepeatMasker
TE: transposable element.

## 1 INTRODUCTION

Besides genes, plant genomes contain intergenic sequences, which have increasing repetitive sequences as the genome size grows. The growth in repeat content is roughly linear up to a genome size of 10 Gbp, including most known angiosperms, and then plateaus (Novák et al., 2020). The repetitive fraction of the genome is made up of low‐copy repeats, simple repeats (such as satellite DNA), and transposable elements (TEs), which were discovered by Barbara McClintock in maize (*Zea mays* L.) (McClintock, 1950).

Transposable elements can be important for explaining observed phenotypes or domestication [see, for instance, Studer et al. (2011)] and are used as a source of genetic variability in breeding programs (Thieme et al., 2017). The hypothesis is that the copy‐and‐paste and cut‐and‐paste mechanisms of TEs might leave footprints in the genome and can potentially affect the expression, regulation, or coding sequences of neighboring genes. Moreover, TEs are increasingly receiving attention in studies tackling plant pangenomes (e.g., Gordon et al., 2017). According to the Wicker classification, plant TEs can be classified either as Class I RNA retrotransposons or Class II DNA transposons (Wicker et al., 2007). Software resources such as RepeatMasker (RM) (Smit et al., 2015), RepBase (Bao et al., 2015), or RepetDB (Amselem et al., 2019), which are typically used to annotate TEs and other repeats in plant genomes, use the Wicker classification rules and repeat libraries (Lerat, 2010). These libraries can be generic, such as RepBase, which is available for subscribers only, or customized for a genome of interest with RepeatModeler (RepMod) (Flynn et al., 2020). These repeat annotation strategies can take up to several days on a computer cluster, depending on the genome size, and often mask disease resistance (R) genes, which are of great interest in plant breeding (Bayer et al., 2018).

In addition to the intrinsic biological value of TEs, the annotation of repeats can be used to estimate assembly quality (Wierzbicki et al., 2020) as an alternative to gene completeness (Van Bel et al., 2019). For other genomic analyses, the bulk of repeated sequences may disrupt common computational genomic analyses and are thus often masked out, without any classification attempt. For instance, whole‐genome alignment, promoter analysis, and the construction of graph genomes require the computation of frequency tables of k‐mers, which are nucleotide words of size *k*. If repeated sequences are not masked, the frequency tables are severely biased and can affect the results obtained (Hickey et al., 2020). Although annotation approaches based on sequence similarity are computationally expensive, k‐mer masking strategies are orders of magnitude faster (Beier et al., 2020; da Cruz et al., 2020; Girgis, 2015; Kurtz et al., 2008) and, in our experience, are much better for prepare whole‐genome alignments of barley (*Hordeum vulgare* L.) and wheat (*Triticum aestivum* L.) cultivars via LASTZ (Harris, 2007).

In this study, we benchmarked a two‐step approach for annotating repeated sequences in plants. First, repeats were called by k‐mer counting with the Repeat Detector (Red). Second, the discovered repeated sequences were annotated by sequence alignment to a newly curated metacollection of repeats called nrTEplants. We compared this approach with the conventional RM pipeline on a set of 20 angiosperms from Ensembl with nrTEplants, REdat (Nussbaumer et al., 2013) and custom RepMod libraries. We then compared their performance and discuss the results. The nrTEplants library is bundled with documentation on how to update it and scripts to mask and annotate plant genomes, enabling interoperability, reuse, and reproducible analyses (Wilkinson et al., 2016).

**Core Ideas**

- Control Pfam domains minimize unrelated coding sequences in repeat libraries.
- Repeat calling by k‐mer counting with Red does not preferentially mask NLR genes.
- Repeats called by Red can be efficiently classified by sequence similarity with minimap2.

## 2 MATERIALS AND METHODS

### 2.1 Plant repeat libraries

We searched the literature for plant‐specific libraries of repeated sequences and selected those in Table 1. Although some are specific for a species or repeat family, others comprise repeats from mixed species, such as REdat from PlantsDB (Nussbaumer et al., 2013) or RepetDB (Amselem et al., 2019). FASTA files with nucleotide sequences of repeats were downloaded from the indicated URLs or obtained from the authors.

**TABLE 1:** Collections of plant repeated sequences used as components of nrTEplants

**Dataset**	**Description and source**	**Last updated**	**Total sequences**	**Median length**
				bp
TREP	TEs^a^ from Triticeae and various other species. https://botserv2.uzh.ch/kelldata/trep‐db/index.html	2019	4,162	4,234
SINEbase	Consensus sequences of Short interspersed nuclear element families (Vassetzky & Kramerov, 2013). http://sines.eimb.ru	2020	60	183
REdat	Repeats from several sources and species in PlantsDB (Nussbaumer et al., 2013). https://pgsb.helmholtz‐muenchen.de	2013	61,730	7,504
RepetDB	Repeats detected and classified by TEdenovo and used by TEannot (Amselem et al., 2019). http://urgi.versailles.inra.fr/repetdb	2019	33,416	3,567
EDTArice	Extensive de novo TE Annotator (Ou et al., 2019). https://github.com/oushujun/EDTA	2019	2,431	984
EDTAmaize	Extensive de novo TE Annotator (Ou et al., 2019) https://github.com/oushujun/EDTA	2019	1,362	3,308
SoyBaseTE	Comprehensive database of soybean TEs (Du et al., 2010). https://www.soybase.org/soytedb	2010	38,664	1,716
TAIR10TE	*Arabidopsis thaliana* TEs https://www.arabidopsis.org	2019	31,189	305
SunflowerTE	Staton et al. (2012) https://www.sunflowergenome.org	2016	73,627	4,709
SUNREP	The repetitive component of the sunflower genome (Natali et al., 2013) pgagl.agr.unipi.it/sequence‐repository	2013	47,441	616
MelonTE	Castanera et al. (2019)	2020	1,560	3981
RosaTE	Hibrand Saint‐Oyant et al. (2018) https://iris.angers.inra.fr/obh/downloads	2017	355,304	226

^a^ TE, transposable element.

### 2.2 Plant transcript sequences

Plant species in Ensembl Plants release 46 (November 2020) (Howe et al., 2020) were ranked in terms of the number of proteins reviewed in Uniprot on 22 Feb. 2020 (UniProt Consortium, 2019). This was considered as an indicator of annotation quality, as UniProt protein sequences are commonly used during prediction and validation of gene models. A list of the best‐annotated dicot and monocot species was produced, including *Arabidopsis thaliana* (L.) Heynh., *Brassica napus* L., *Glycine max* (L.) Merr., sunflower (*Helianthus annuus* L.), *Medicago truncatula* Gaertn.*, Phaseolus vulgaris* L.*, Populus trichocarpa* Torr. & A.Gray ex Hook., *Solanum lycopersicum* L., *Vitis vinifera* L., *Brachypodium distachyon* (L.) P.Beauv.*, Hordeum vulgare, Oryza sativa* subsp. *japonica* L., *Sorghum bicolor* (L.) Moench., and *Zea mays*. Transcripts (cDNA) from these species were downloaded with the script *ens_sequences.pl* from https://github.com/Ensembl/plant‐scripts.

### 2.3 Sequence clustering

Transcripts and TE sequences were clustered with GET_HOMOLOGUES‐EST version 10042020 (Contreras‐Moreira et al., 2017). This software runs BLASTN and the MCL algorithm, and computes coverage by combining local alignments. The sequence identity cut‐off was 95% and the alignment coverage 75%. Global variables in the script *get_homologues‐est.pl*, lines L36‐7, were set to $MAXSEQLENGTH = 55000 and $MINSEQLENGTH = 90. Sequences were clustered with the command *get_homologues‐est.pl ‐d repeats ‐m cluster ‐M ‐t 0 ‐i 100*. The longest sequence in each cluster was taken as a representative.

### 2.4 Positive control Pfam domains

A list of 22 Pfam domains found in TEs was curated (Mistry et al., 2021), available at https://github.com/Ensembl/plant_tools/blob/master/bench/repeat_libs/control_pos.list.

### 2.5 Negative control: Pfam domains of disease resistance genes

For the identification and curation of Pfam domains encoded by disease resistance (R) genes, the following steps were performed. First, a set of 153 protein sequences encoded by reference R genes (i.e., cloned and/or with robust evidence) was retrieved from http://www.prgdb.org/prgdb (Osuna‐Cruz et al., 2018). Second, the program *hmmscan* from HMMER Version 3.2.1 (Eddy, 1998) was used for initial Pfam domain identification (Version 32, default settings), yielding a total of 60 Pfam hidden Markov models. The observed order and combinations of Pfam domains were retrieved. Third, the proteins of six plant species (*A. thaliana, B. distachyon, G. max, H. annuus, H. vulgare*, and *T. aestivum*) containing at least one of the 60 Pfam domains previously identified were retrieved from https://plants.ensembl.org/biomart/martview (Kinsella et al., 2011). These proteins were subsequently filtered, retaining only those with the ordered combinations of Pfam domains observed in the reference R proteins, and were considered as potential R proteins (428 in *A. thaliana*, 577 in *B. distachyon*, 1,008 in *G. max*, 849 in *H. annuus*, 838 in *H. vulgare*, and 3,607 in *T. aestivum*). From the initial set of Pfam domains, only 43 were consistently identified in our final panel of potential encoded proteins of R genes and used as a negative control. Note that one of them (PF02892, zf‐BED) is often found in transposases (Mistry et al., 2021). The list is available at https://github.com/Ensembl/plant_tools/blob/master/bench/repeat_libs/control_neg_NLR.list.

### 2.6 De novo annotation of nucleotide‐binding and leucine‐rich repeat immune receptor genes

The NLR‐annotator software package (Steuernagel et al., 2020) was used for *de novo* annotation of nucleotide‐binding and leucine‐rich repeat immune receptor (NLR) genes, which are the most abundant R genes characterized to date, in whole genome sequences. Briefly, the 20 plant genomes were dissected into fragments 20 kb in length, with 5 kb overlaps, via the *ChopSequence.jar* routine. The cut sequences were then scanned to find NLR‐associated sequence motifs with the *NLR‐Parser.jar* command. Finally, *NLR‐Annotator.jar* was used to integrate the annotated motifs and retrieve the actual NLR loci in BED format. In order to compute intersections with repeats, only NLR loci with an overlap of  >50 bp were considered. Moreover, to account for the fact that the tested masking strategies covered different fractions of the genome, odd ratios of NLR masking were computed via Equation 1:

(1) $$\mathrm{OR}=\frac{{\mathrm{NLR}}_{\mathrm{masked}}÷{\mathrm{Gen}}_{\mathrm{masked}}}{\mathrm{NLR}÷\mathrm{Gen}},$$

where OR is the odds ratio, NLR_masked_ is the masked NLR space, Gen_masked_ is the masked genome space, NLR is the NLR space, and Gen is the genome space.

### 2.7 Masking and annotation of repeats in plant genomes

RepeatMasker Version 4.0.5 and a fork of Repeat Detector (Red) Version 2.0 adapted for Ensembl, available at https://github.com/EnsemblGenomes/Red, were used to call repeats in plant genomes in the libraries REdat Version 9.3 and nrTEplanst Version 0.3. In addition, RepeatMasker Version 4.1.2‐p1 was also run to call repeats with custom repeat libraries produced by 20 parallel jobs in RepeatModeler‐2.0.2a (Flynn et al., 2020). Note that custom libraries were obtained for only 10 species, as the remaining RepMod jobs were killed after 7 d in a computer farm. RepMod repeat coordinates were converted to BED format and overlapping intervals were merged. Low complexity sequences were called with dustmasker Version 1.0.0 (Morgulis et al., 2006). Tandem repeats were discovered with trf Version 4.0 with the parameters *2 5 7 80 10 40 500 ‐d ‐h* (Benson, 1999). Red was called from the script https://github.com/Ensembl/plant-scripts/blob/master/repeats/Red2Ensembl.py, which can run several sequences in parallel and feed the results into a Ensembl core database (Stabenau et al., 2004). In addition, minimap2 version 2.17‐r974‐dirty (Li, 2018) was used to annotate the repeats called by Red with sequences from nrTEplants as follows: *minimap2 ‐K100M –score‐N 0 ‐x map‐ont nrTEplants*. Minimap2 is called from the script https://github.com/Ensembl/plant-scripts/blob/master/repeats/AnnotRedRepeats.py, which parses its output to annotate the repeats. By default, only repeats with a length of >90 bp are processed. Transposable element classification terms are parsed from the FASTA header of the library after a hash (#; e.g., RLG_43695:mipsREdat_9.3p_ALL#LTR/Gypsy). Elapsed runtime and RAM consumption was measured with the *command time ‐v* tool.

Genomic intersections among repeated sequences called by Red and RM, and genomic features (i.e., protein‐coding genes, exons, proximal downstream and upstream 500‐bp windows, and NLR loci) were computed with Bedtools (Version 2.26.0) (Quinlan & Hall, 2010) using *bedtools intersect ‐a bed/genes.bed ‐b repeat.bed ‐sorted ‐wo*. To avoid redundancy, exons were extracted from Ensembl canonical transcripts (see http://plants.ensembl.org/info/website/glossary.html). When we retrieved downstream and upstream genomic intervals, intersecting neighbor genes were first subtracted to eliminate any potential coding sequences.

### 2.8 K‐mer analysis of repeats in downstream and upstream windows

Repeats overlapping proximal downstream or upstream 500‐bp windows were extracted via *bedtools intersect* analysis and the sequences were cut with *bedtools getfasta*. Canonical k‐mers with k = [16,21,31] were counted with Jellyfish Version 2.3.0 (Marçais & Kingsford, 2011) by the commands *jellyfish‐linux count ‐C ‐m K ‐s 2G ‐t 4* and *jellyfish‐linux dump ‐L 20*.

### 2.9 Enrichment of Pfam domains

Enrichment was computed by the R function fisher.test (R Core Team, 2020) and Pfam domains (Mistry et al., 2021) were retrieved by Recipe B4 of https://github.com/Ensembl/plant‐scripts (Contreras‐Moreira et al., 2021). Pfam domain counts for the complete proteome were used as the expected frequencies. Only genes with an overlap of >50 bp and domains with adjusted false discovery rates (*p* < .05) were considered.

### 2.10 Control sets of annotated repeated sequences

Repeated sequences annotated by the sequencing consortia of olive tree (*Olea europaea* L.)(Jiménez‐Ruiz et al., 2020), *Rosa chinensis* Jacq. (Hibrand Saint‐Oyant et al., 2018), and sunflower (Badouin et al., 2017) were downloaded from https://genomaolivar.dipujaen.es/db/downloads.php, https://iris.angers.inra.fr/obh/downloads, and https://sunflowergenome.org/annotations‐data, respectively.

## 3 RESULTS AND DISCUSSION

### 3.1 Construction and benchmarking of a nonredundant library of repeats: nrTEplants

A set of plant TE libraries and annotated repeats from selected species, listed in Table 1 plus transcript sets from the best functionally annotated plant species in Ensembl were curated and their TE classification terms uniformized. Next, they were merged and clustered (95% identity, 75% coverage of shortest sequence). From the resulting 994,349 clusters, the 174,426 clusters contained TE sequences and were six‐frame translated and assigned Pfam domains. Of these, a subset of 8,910 mixed clusters comprising both TE and transcript sequences, and required further processing (see the example in Supplemental Figure S1). After empirical assessment, we decided to take only clusters (a) containing sequences from at least six different TE libraries (six replicates), which eventually left out *Rosa* TE repeats; and (b) those with a fraction of sequences marked as a ‘potential host gene’ in RepetDB below 0.00. The resulting nrTElibrary contained 171,104 sequences (see Supplemental Table S1 and Supplemental Table S2). Note that different cut‐off values might have been selected with different input sequences or control sets. For example, increasing the number of replicates equates to computing an intersection set. Instead, to get a union set, the cut‐off will need to be lowered.

In order to benchmark the newly constructed library, we compiled a positive control comprising 22 Pfam domains found in TEs, and a negative control: a list of 43 Pfam domains found in disease resistance NLR genes. Among these controls, we observed 20 true positives, 2 false negatives, 36 true negatives, and 2 false positives, yielding a sensitivity of 0.91 and a specificity of 0.95. The nrTEplants library can be obtained at https://github.com/Ensembl/plant‐scripts/releases/tag/v0.3. A step‐by‐step guide on how to produce a nonredundant repeat library, including sample files with the control Pfam domains, is available at https://github.com/Ensembl/plant_tools/tree/master/bench/repeat_libs.

### 3.2 Masking repeats within plant genomes

Twenty plant genomes were selected from Ensembl (Howe et al., 2020) to benchmark the repeat calling strategies. These are listed in Table 2 next to the genomic fraction of repeats reported in the literature and their guanine–cytosine content. All these genome sequences were annotated with RM (Smit et al., 2015) with several repeat libraries (nrTEplants and REdat) (Nussbaumer et al., 2013) and species‐specific custom libraries (RepMod). In addition, the fraction of repeats called by Red, based on k‐mer enrichment, is also shown. Note that Red automatically selected k values from 13 to 16 as the genomes increased in length.

**TABLE 2:** Plant genomes from release 49 (September 2020) of Ensembl Plants (Howe et al., 2020) used in this work and their reported repeated fractions in the literature

**Species**	**GC** ^a^	**Assembled genome size**	**Reported repeated fraction**	**Literature source**
	%	Mbp	%	
*Arabidopsis thaliana*	36.1	119.7	19.0	Legrand et al. (2019)
*Arabidopsis helleri* (L.) O'Kane & Al‐Shehbaz	36.0	196.2	32.7	Legrand et al. (2019)
*Prunus dulcis* (Mill.) D.A.Webb	37.6	227.5	37.6	Alioto et al. (2020)
*Brachypodium distachyon*	46.4	271.2	21.4	International Brachypodium Initiative (2010)
*Brassica rapa* L.	35.3	283.8	32.3	Zhang et al. (2018)
*Trifolium pratense*	32.4	304.8	41.8	De Vega et al. (2015)
*Arabis alpina* L.	36.8	308.0	47.9	Willing et al. (2015)
*Cucumis melo* L.	33.5	357.9	44.0	Ruggieri et al. (2018)
*Citrullus lanatus* (Thunb.) Matsum. & Nakai	33.6	365.5	45.2	Guo et al. (2013)
*Oryza sativa*	43.6	375.0	35	International Rice Genome Sequencing Project (2005)
*Setaria viridis* (L.) P.Beauv.	46.2	395.7	46	Thielen et al. (2020)
*Vitis vinifera*	34.5	486.3	41.4	French–Italian Public Consortium for Grapevine Genome Characterization (2007)
*Rosa chinensis*	38.8	515.6	67.9	Raymond et al. (2018)
*Camelina sativa* (L.) Crantz	36.6	641.4	28	Kagale et al. (2014)
*Malus domestica* Borkh.	38.0	702.9	59.5	Daccord et al. (2017)
*Olea europaea*	35.4	1,140.9	43	Unver et al. (2017)
*Zea mays*	46.9	2,135.1	85	Schnable et al. (2009)
*Helianthus annuus*	38.5	3,027.8	74.7	Badouin et al. (2017)
*Aegilops tauschii*	46.3	4,224.9	85.9	Zhao et al. (2017)
*Triticum turgidum*	46.0	10,463.1	82.2	Maccaferri et al. (2019)

^a^ GC, guanine–cytosine content

**FIGURE 1:**
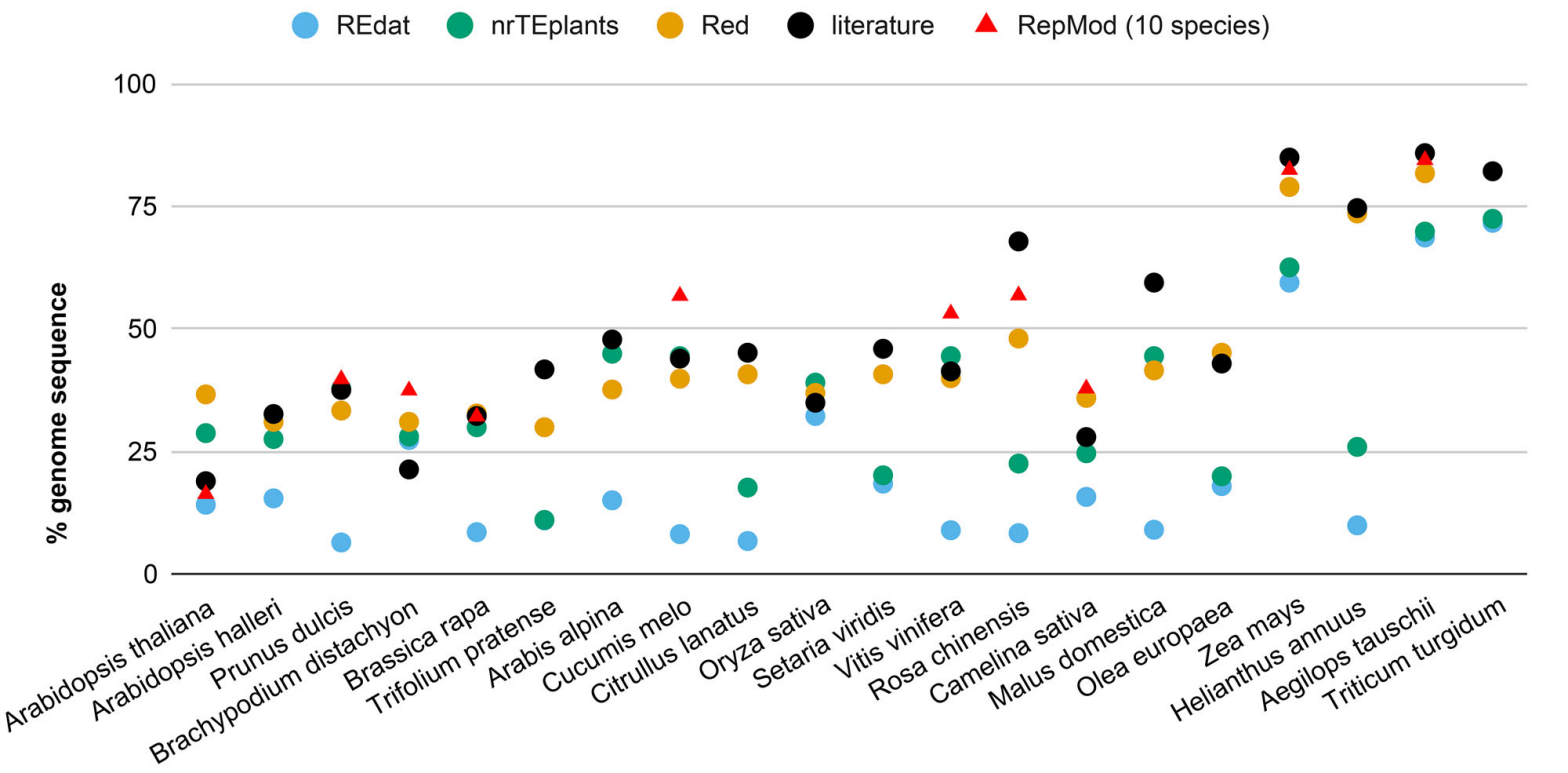
Fraction of repeated sequences in plant genomes. Twenty genomes from release 49 (November 2020) of Ensembl Plants were annotated with RepeatMasker (Smit et al., 2015) and the libraries REdat (Nussbaumer et al., 2013) and nrTEplants. The results for 10 genomes masked with RepMod custom libraries are also shown (Flynn et al., 2020). The percentage of repeated sequences is plotted next to the values reported in the literature for those genomes and the fraction of repeats provided by Repeat Detector (Red), based on k‐mer enrichment (Girgis, 2015). Species are sorted by genome size from smallest to largest

In Figure 1, the resulting percentages of repeated sequences are plotted next to the values reported in the literature. The median difference between the REdat repeated fraction and the literature reports is 26.5%. This number is 9.8% for nrTEplants, 4.3% for Red, and 6.3% for RepMod (over 10 genomes). These results suggest that Red can successfully mask any genomes without previous knowledge of the repetitive sequence repertoire of a species. As shown in Supplemental Table S3, Red‐masked fractions were also consistent among cultivars of the wheat pangenome. Moreover, repeats called by Red generally overlapped sequences masked with REdat (66.6%), nrTEplants (73.8%), and RepMod (94.1%) (see Supplemental Table S4). In contrast, the overlap with low complexity regions (in dustmasker) and tandem repeats (in trf) is small (2.8% and 4.9%, respectively).

Table 3 summarizes the number and length of repeats called by all the strategies tested. We observed that Red called more repeats than nrTEplants and REdat but less than custom RepMod libraries (a median of 845 per Mbp, compared with 391 for nrTEplants, 221 for REdat, and 961 for RepMod). In terms of the sequence length of the shortest contig at 50% of the total sequence length, the performance depended on the species, but it seems that repeats called by RepMod are generally shorter.

**TABLE 3:** Summary of repeated sequences annotated with Repeat Detector (Red) (Girgis, 2015) and RepeatMasker (Smit et al., 2015) with the libraries nrTEplants and REdat (Nussbaumer et al., 2013) and with custom libraries obtained for some species by RepeatModeler (Flynn et al., 2020). Total repeats and N50 is the sequence length of the shortest contig at 50% of the total sequence length (N50) estimates of repeats are shown

	**Red**		**nrTEplants**		**REdat**		**RepMod**	
**Species**	**Repeats**	**N50**	**Repeats**	**N50**	**Repeats**	**N50**	**Repeats**	**N50**
*Arabidopsis thaliana*	172,935	445	48,144	1,779	28,797	2,211	72,138	1,178
*Arabidopsis halleri*	226,080	554	81,857	1,380	57,901	1,431	–	–
*Prunus dulcis*	190,357	1,627	105,546	2,528	36,891	1,025	243,499	1,422
*Brachypodium distachyon*	150,191	4,986	74,215	6,260	67,632	6,665	222,710	2,125
*Brassica rapa*	348,258	642	160,157	1,046	69,345	777	303,119	628
*Trifolium pratense*	277,811	555	139,254	326	155,808	265	–	–
*Arabis alpina*	279,129	1,040	146,057	2,245	98,017	1,050	–	
*Cucumis melo*	305,083	1,939	148,925	3,141	51,833	1,338	407,579	1,819
*Citrullus lanatus*	323,894	2,596	151,980	1,020	52,941	1,103	–	–
*Oryza sativa*	278,406	2,931	160,371	4,479	129,121	6,077	–	–
*Setaria viridis*	247,732	3,124	116,459	1,727	105,088	1,722	–	–
*Vitis vinifera*	423,876	1,753	185,204	3,369	69,315	1,550	496,352	1,604
*Rosa chinensis*	463,880	2,125	189,086	1,479	93,715	950	499,475	1,958
*Camelina sativa*	709,160	878	267,290	1,272	201,059	1,176	611,700	1,105
*Malus domestica*	531,496	2,416	211,929	4,729	126,487	1,268	–	–
*Olea europaea*	901,519	3,153	291,445	1,956	375,614	1,218	–	–
*Zea mays*	847,205	13,137	365,978	11,806	372,467	11,419	853,432	1,1380
*Helianthus annuus*	2,387,122	5,018	355,890	8,716	479,400	1,317	–	–
*Aegilops tauschii*	1,506,690	10,133	777,962	9,973	847,592	9,431	1,758,407	7,894
*Triticum turgidum*	4,291,533	9,066	1,914,776	9,947	1,784,719	10,124	72,138	1,178

**FIGURE 2:**
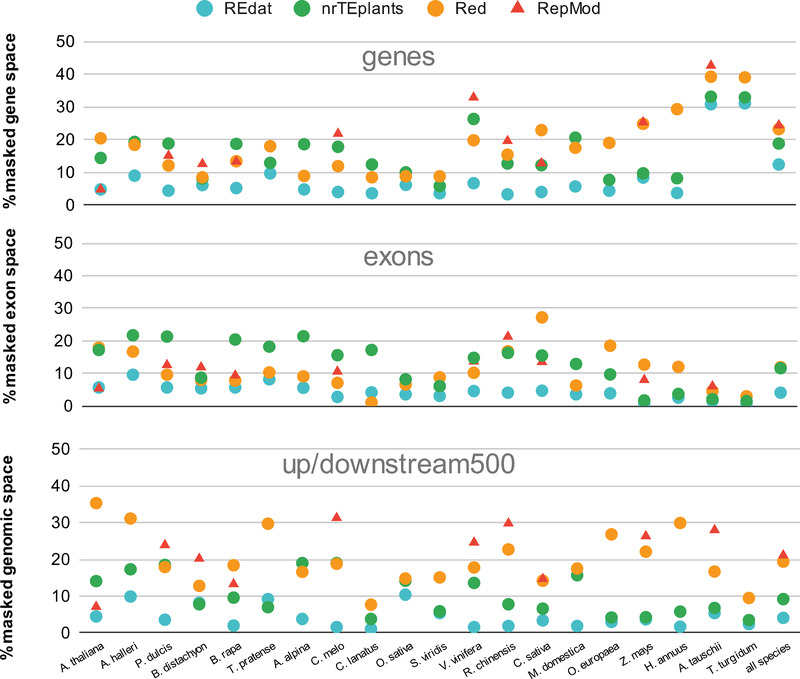
Fraction of exons, genes, and 500‐bp upstream and downstream regions overlapping annotated repeats in plant genomes. Twenty genomes from release 49 (November 2020) of Ensembl Plants were annotated by Red (Girgis, 2015) or RepeatMasker (Smit et al., 2015) with the libraries REdat (Nussbaumer et al., 2013) and nrTEplants. The results for 10 genomes masked with RepMod custom libraries are also shown (Flynn et al., 2020)

Figure 2 summarizes how the called repeats overlapped with genes, exons, and 500‐bp windows upstream and downstream. It can be seen that Red repeats overlapped a larger fraction of the gene space (23.2%) than REdat (12.4%) and nrTEplants (18.8%), as did RepMod repeats (24.4%). When only exons were considered, REdat repeats overlapped 4.1% of these, with nrTEplants, Red, and RepMod behaving similarly (11.6, 11.9, and 11.7%, respectively). The figure also shows that Red and RepMod mask more of the proximal upstream and downstream space, which will probably have a positive impact on k‐mer counting strategies for promoter analysis (Ksouri et al., 2021). The analysis in Supplemental Table S5 shows that Red identified four times more k‐mers with 20+ copies in this regulatory space, which agrees with recent work showing that unidentified TEs are over‐represented in specific regulatory networks (Baud et al., 2019).

**FIGURE 3:**
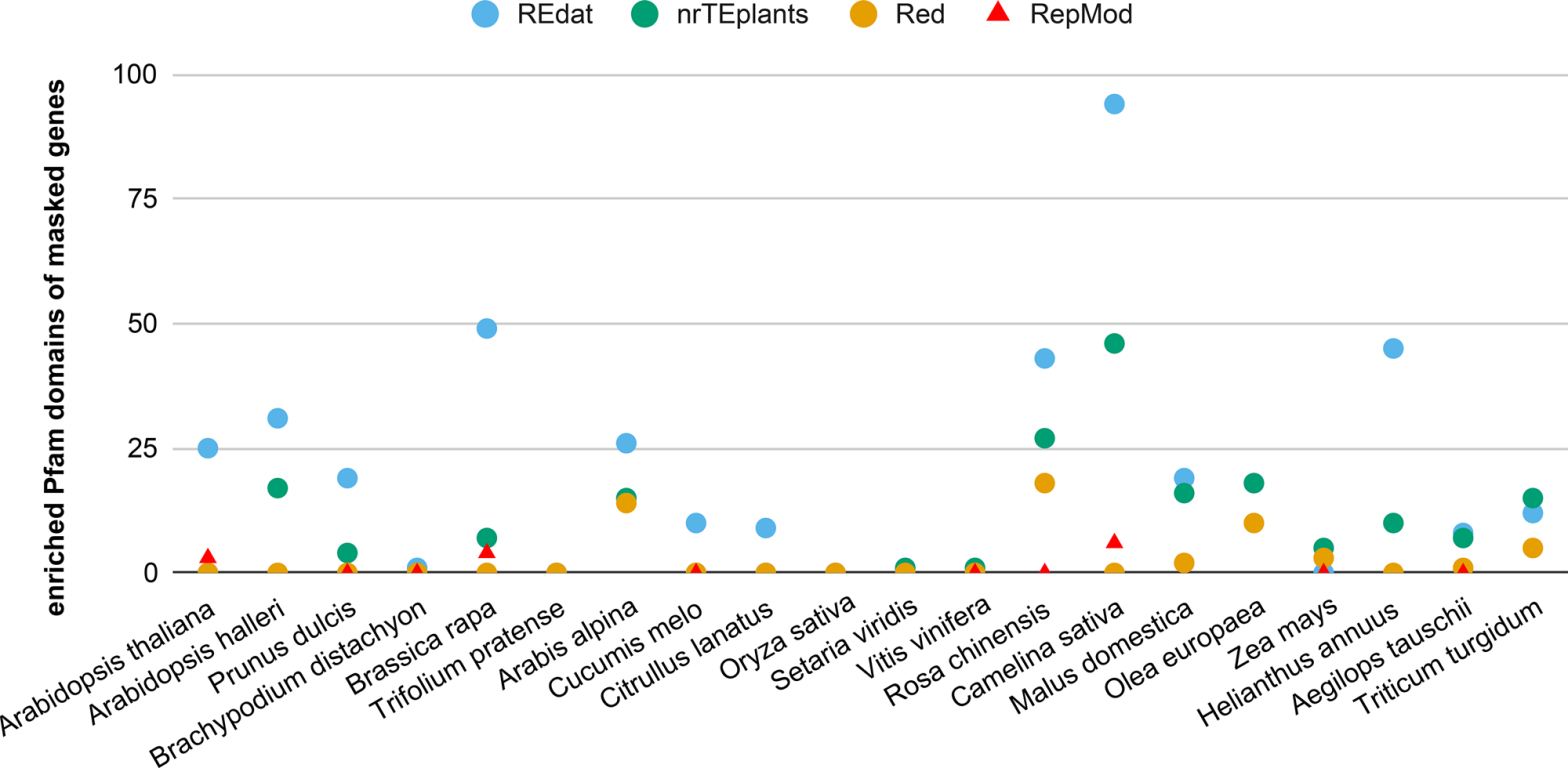
Enriched Pfam domains of protein‐coding genes overlapping repeats. Twenty genomes from release 49 (November 2020) of Ensembl Plants were annotated with Repeat Detector (Red) (Girgis, 2015) and RepeatMasker (Smit et al., 2015) with the libraries REdat (Nussbaumer et al., 2013) and nrTEplants. The results for 10 genomes masked with RepMod custom libraries are also shown (Flynn et al., 2020)

In order to check whether the compared approaches masked preferentially genes from certain families, a Pfam enrichment analysis was carried out; this is summarized in Figure 3. It can be seen that RepMod and Red repeats show the least enrichment. Nevertheless, we found that Red repeats preferentially overlapped four domains (enriched in three or more genomes: reverse transcriptase‐like, TIR, NB‐ARC, and integrase core domains). Similarly, RepMod repeats were enriched in two protein kinase domains. In contrast, a few Pfam domains were enriched in 10+ genomes in genes overlapping repeats annotated with REdat (153 domains) and nrTEplants (87 domains)(see Supplemental Table S6).

As gene annotation is frequently performed after repeat masking, we reasoned this could affect the Pfam enrichment analyses. Therefore, we carried out a complementary analysis where NLR genes were called de novo on the genomic sequences instead of using the Ensembl gene annotation. The results, summarized in Supplemental Table S7, confirm that Red tends to mask fewer NLR genes than expected at genomic scale, with only one species (*Trifolium pratense* L.) with an odds ratio >1. In contrast, we obtained odd ratios greater than 1 for several species with REdat (*n* = 7), nrTEplants (*n* = 12), and RepMod (*n* = 6 out of 10 species).

### 3.3 Annotating Red‐masked repeats within genomes with nrTEplants and minimap2

**FIGURE 4:**
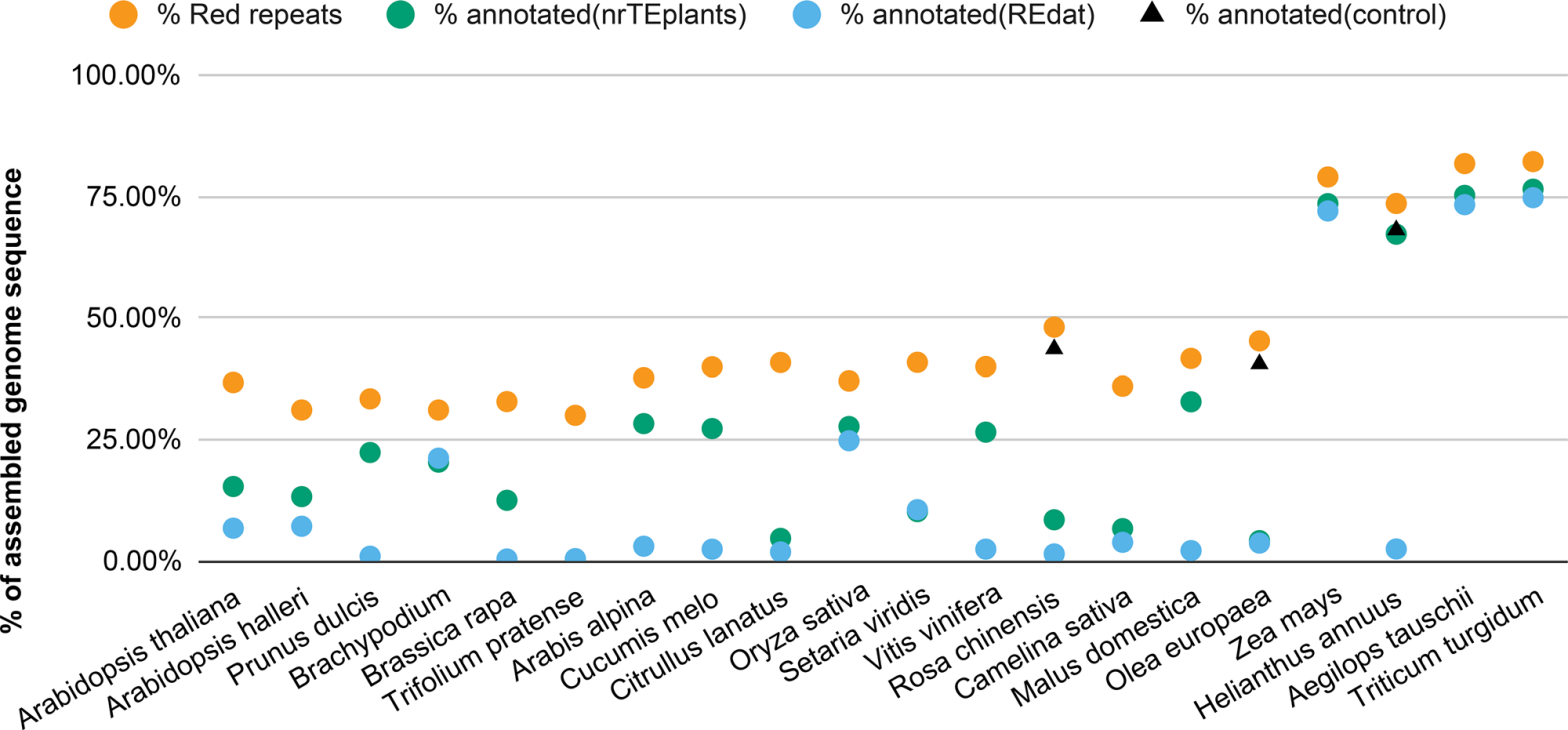
Fraction of Repeat Detector (Red) repeats mapped to nrTEplants sequences. Twenty genomes from release 49 (November 2020) of Ensembl Plants were annotated with Red (Girgis, 2015). The resulting repeats were subsequently mapped to the library nrTEplants with minimap2 (Li, 2018), producing the genome fractions shown. Repeats from three species (*R. chinensis, O. europaea*, and *H. annuus*) were also mapped to annotated repeats provided by the respective sequencing consortia as a control. Species are sorted by genome size from smallest to largest

In the previous analyses, we showed that Red masking is an effective way of calling repeats in plant genomes, comparable with RepMod. Moreover, we observed that nrTEplants behaved better than REdat in most cases. Therefore, we wanted to check whether repeats called with Red could be annotated and classified. For that, we aligned the repeat sequences against the nonredundant nrTElibrary with minimap2. The results are plotted in Figure 4, where it can be seen that in most species, more than half of the repeat space could be annotated (median: 65.9%). As our library contained only TEs, we expected a fraction of the unmapped space to contain simple repeats or satellite DNA. However, in some species, only a small fraction of repeats could be classified. We reasoned this was caused by a repeat consensus not represented in the library. This was confirmed in a separate experiment, where the repeated sequences of olive and *R. chinensis* obtained from their authors were mapped to Red repeats, as seen in Figure 4 (control). Another positive control was also carried out with sunflower repeated sequences in order to confirm that no valuable repeats had been lost during the construction of nrTEplants. These results indicated that in species where a curated library did not work well, the repeats could be classified by custom collection of repeated sequences for that taxon. As we saw in the previous section, this can also be achieved with species‐specific libraries produced with RepMod; however, note that in our tests three‐fourths of repeat families discovered by RepMod remained unclassified (see Supplemental Table S8).

The results in the previous paragraph were obtained with the default *map‐ont* setting of minimap2. Note that we also tried the *map‐pab* and *asm20* settings, but obtained similar results. Red clover (*Trifolium pratense*) was reanalyzed replacing minimap2 with the BLAST algorithms *megablast*, *dc‐megablast*, *blastn*, and *rmblastn* (Altschul et al., 1997). Compared with the mapped fraction produced by minimap2 (0.4%), a maximum value of 6.1% was obtained with *blastn*. This modest gain in sensitivity required 1,412 min. The algorithm *rmblastn*, used by RM, yielded a mapped fraction of 0.7%. We concluded that the alternatives to minimap2 offered little gain at the cost of spiralling computing time.

**FIGURE 5:**
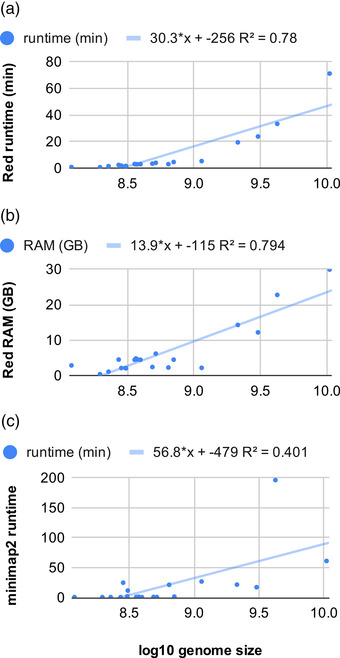
Runtime and memory requirements of a two‐step repeat annotation protocol based on the Repeat Detector (Girgis, 2015), minimap2 (Li, 2018), and the nrTEplants library. The protocol was tested on 20 genomes from release 49 (November 2020) of Ensembl Plants. Similar values were measured on an Ubuntu box with four‐core i5‐6600 (3.30 GHz) central processing unit cores

Figure 5 shows the runtime and RAM required by the two‐step protocol presented in this paper, measured on a CentOS7.9 computer using four cores of a Xeon E5‐2620 v4 (2.10 GHz) central processing unit. Panels A and B correspond to the first step, Red masking. It can be seen that all genomes tested take less than 40 min to run, with the exception of tetraploid *Triticum turgidum* L., which took 71 min. The memory consumption was below 20 GB in most cases, but climbed to 22.7 GB and 29.9 GB for *Aegilops tauschii* Coss. and *T. turgidum*. Panel C illustrates the runtime of the second step, the mapping of nrTEplants. It can be seen that all plants required less than 27 min, except *A. tauschii* and *T. turgidum*, which took 3 and 1 h respectively. The memory consumed by minimap2 was ∼3.8 GB in all cases. A comparison with the data in Supplemental Table S8 indicated that the protocol presented in this paper was up to two orders of magnitude faster than the combination of RepMod and RM, even with only four central processing unit cores.

## 4 CONCLUSIONS

The hybrid two‐step methodology presented in this paper was tested on 20 angiosperms with genome sizes ranging from 0.12 to 10.46 Gbp. Overall, we observed that Red consistently produced repeated fractions similar to the expected values from the literature. Comparable results were obtained for 10 species analyzed with RepMod custom libraries. The meta‐library nrTEplants, built by Pfam‐informed sequence clustering, also showed good performance in most species but failed to recover the expected repeat fraction in cases such as melon (*Cucumis melo* L.) or sunflower. This observation highlights the problem of using repeat libraries that do not include sequences similar to the genome of interest. This is the most likely explanation for the low masking values observed for REdat, as that library was produced before many of these genomes were available. For that reason, separating the tasks of calling and classifying repeats, as performed here, seems a promising strategy.

On the one hand, Red k‐mer masking does not have a preference for masking particular protein‐coding families, in contrast to repeats annotated with RM using REdat and nrTEplants. In fact, it also behaved better than custom RepMod libraries with respect to NLR genes annotated de novo . On the other hand, Red appropriately masked plant genomes for which no repeat libraries have been curated yet. If there is a need to classify the repeats called by Red, a curated repeat library can be obtained directly from Ensembl Plants (see https://github.com/Ensembl/plant‐scripts/blob/master/repeats/get_repeats_ensembl.sh) or the INSDC archives (see, for example, https://www.ebi.ac.uk/ena/browser/view/CACTIH01), or by clustering repeats from different sources, as demonstrated in this study. Our protocol took less than 2 h to run and up to 30 GB of RAM, and can use nrTEplants or any repeat library in FASTA format. This is about two orders of magnitude faster than building species‐specific custom libraries with RepMod for the species tested in this benchmark. We thus conclude that the approach presented here is an efficient way of annotating repeated sequences in plant genomes.

## DATA AND SOURCE CODE AVAILABILITY

The repeat library and the scripts used to mask and annotate the plant genomes, together with the benchmark scripts and data, can be obtained at https://github.com/Ensembl/plant‐scripts.

## AUTHOR CONTRIBUTIONS

Bruno Contreras‐Moreira: formal analysis, funding acquisition, investigation, resources, software, supervision, writing—original draft, writing—review and editing. Carla V Filippi: data curation, investigation, methodology, resources, writing—original draft, writing—review and editing. Guy Naamati: resources, software, writing—review and editing. James E Allen: resources, software, writing—review and editing. Paul Flicek: funding acquisition, resources, writing—original draft, writing—review and editing.

## CONFLICT OF INTEREST

Paul Flicek is a member of the Scientific Advisory Boards of Fabric Genomics, Inc. and Eagle Genomics Ltd.

## Supporting information


**Supplemental Figure S1**. Cluster with two *Arabidopsis thaliana* cDNA sequences (AT4G16920.2 and AT4G16950.1) and the TE TEdenovo‐B‐R2288‐Map4 from library RepetDB. These sequences contain the Pfam domain NB‐ARC (PF00931), which is part of the NLR defense proteins. The figure was generated with Bioedit (Hall, 1999) from the cluster 269_AT4G16920.2.

Supplemental Table S1. Contribution of individual datasets to the nrTEplants library of repeats (Version 0.3).
Supplemental Table S2. Summary of classified repeats in the nrTEplants library.
Supplemental Table S3. Wheat cultivars from Walkowiak et al. (2020) annotated with Red (Girgis, 2015).
Table S4. Intersection percentages among repeats called by Red (Girgis, 2015) and repeated sequences called by RM (Smit et al., 2015) with nrTEplants, REdat (Nussbaumer et al., 2013), custom RepMod libraries (Smit et al., 2015), dustmasker (Morgulis et al., 2006), and trf (Benson, 1999).
Supplemental Table S5. Median number of repeat k‐mers with 20+ copies overlapping 500 bp upstream and downstream regions in plant genomes.
Supplemental Table S6. Enriched Pfam domains of protein‐coding genes overlapping repeats called with Red (Girgis, 2015) and RM (Smit et al., 2015) with the libraries nrTEplants and REdat (Nussbaumer et al., 2013)
Table S7. Odd ratios of NLR masking for genes overlapping >50 bp of masked sequences
Table S8. Repeat families discovered by RepeatModeler (Flynn et al., 2020) after 7 d on a computer cluster with 20 parallel processes on 20 genomes from release 49 (November 2020) of Ensembl Plants. UF, unfinished after 7 d.
