# Supplementary material for: K‐mer counting and curated libraries drive efficient annotation of repeats in plant genomes
Source: Plant Genome. 2021 Sep 25;14(3):e20143. doi: 10.1002/tpg2.20143 (PMC7614178; doi:10.1002/tpg2.20143)
Supplement: Supplementary file 1 [file TPG2-14-e20143-s001.pdf]

```
AT4G16920.2[arabidopsis_thalia  AAAGATGAGTGGGTGAAGATGATGCCTAGGCTTCGAAATGACTCAGATGATAAAATTGAGGAAAC
AT4G16950.1[arabidopsis_thalia  AAAAAAGAGTGGATGGAGATGATGCCTAGGCTCCGAAATGTTTCGGA-GATATTA-TGA--AAAC
TEdenovo-B-R2288-Map4[repetDB.  -----------------------------------------------------------------

                                    1270      1280      1290      1300      1310      1320
AT4G16920.2[arabidopsis_thalia  AATAAAAAAAATAGAGAGTTATTTAAGTGCATTGCATGTTTCTTCAATGGTTTTAAAGTCAGTAA
AT4G16950.1[arabidopsis_thalia  CATCAAAAAGAT-CA-AGATATTTCTTTGCATTGCGTGTTTATTCAATGGTTTTGAGGTCAGTTA
TEdenovo-B-R2288-Map4[repetDB.  -----------------------------------------------------------------

                                    1360      1370      1380      1390      1400      1410
AT4G16920.2[arabidopsis_thalia  GTTGGGCTTACAATGTTGGTTGAGAAGTCCCTCATACGTATTACACCGGATGGAGATATAGAGAT
AT4G16950.1[arabidopsis_thalia  GTTGGGCTTACAATGTTGTCTGAGAAGTCCCTCATACGTATTACACCGGATGGACATATAGAGAT
TEdenovo-B-R2288-Map4[repetDB.  -----------------------------------------------------------------

                                    1450      1460      1470      1480      1490      1500
AT4G16920.2[arabidopsis_thalia  AGAGAAATTGATCGTGCAAAGTCCAAGGGTAATCCTGGAAAACGTCAATTTCTGACAAATTTTGA
AT4G16950.1[arabidopsis_thalia  AGAGAAATTGATCGTGCAAAGTCCAAGGGTAATCCTGGAAAACGTCAATTTCTGACGAATTTTGA
TEdenovo-B-R2288-Map4[repetDB.  -----------------------------------------------------------------

                                    1540      1550      1560      1570      1580      1590
AT4G16920.2[arabidopsis_thalia  AAAACTGGGACAGAAATTCTTCTTGGAATACGTTTGCCACACCCGGGATATCTTACGACAAGGTC
AT4G16950.1[arabidopsis_thalia  AAAACTGGGACAGAAACTCTTCTTGGAATACGTTTGCCATTCGAGGAATATTTTTCGACAAGGCC
TEdenovo-B-R2288-Map4[repetDB.  -----------------------------------------------------------------

                                    1630      1640      1650      1660      1670      1680
AT4G16920.2[arabidopsis_thalia  AAAGGCATGCGTAATCTCCAATATCTAGAAATTGGTTATTGGTCAGATGGGGATCTACCTCAGAG
AT4G16950.1[arabidopsis_thalia  AAAGGCATGCGTAATCTCCAATATCTAAAAATTGGTGATTGGTCAGATGGGGGTCAACCTCAGAG
TEdenovo-B-R2288-Map4[repetDB.  -----------------------------------------------------------------

                                    1720      1730      1740      1750      1760      1770
AT4G16920.2[arabidopsis_thalia  AGATTGCTAGAATGGGTTTATTGTCCGTTGAAGTCTTTGCCATCTACTTTTAGGGCGGAGTATCT
AT4G16950.1[arabidopsis_thalia  AGATTGCTAGACTGGGATGATTGTCCATTGAAGTCTTTGCCATCTACTTTTAAGGCGGAATATCT
TEdenovo-B-R2288-Map4[repetDB.  -----------------------------------------------------------------

                                    1810      1820      1830      1840      1850      1860
AT4G16920.2[arabidopsis_thalia  AAGCTTGAGAAACTGTGGGAAGGAACTCTGCCCCTTGGAAGTCTCAAGAAGATGAATTTGTGGTA
AT4G16950.1[arabidopsis_thalia  AAGCTTGAGAAACTGTGGGAAGGAACTCTGCCCCTTGGAAGTCTCAAGAAGATGAATTTGTTGTG
TEdenovo-B-R2288-Map4[repetDB.  -----------------------------GCCCCTTGGAAGTCTCAAGAAGATGAATTTGTGGTG

                                    1900      1910      1920      1930      1940      1950
AT4G16920.2[arabidopsis_thalia  GATCTTTCTTTAGCCATAAACCTCGAGGAATTAAATCTTTCTGAATGCGAATCTTTGGTGACACT
AT4G16950.1[arabidopsis_thalia  GATCTTTCTAATGCCAGAAACCTCGAGGAATTAGATCTTGAAGGATGCGAATCTTTGGTGACACT
TEdenovo-B-R2288-Map4[repetDB.  GATCTTTCTTTAGCCATAAACCTCGAGGAATTAGATCTTTCTGGATGCGAATCTTTGGTGACACT

                                    1990      2000      2010      2020      2030      2040
AT4G16920.2[arabidopsis_thalia  AAACTGAGGACGTTATATTGTTCGGGGGTGCTATTAATAGATTTAAAATCATTAGAAGGCATGTG
AT4G16950.1[arabidopsis_thalia  AAACTGAGGAAGTTACATTGTTCGGGGGTGATATTAATAGATTTAAAATCATTAGAAGGCATGTG
TEdenovo-B-R2288-Map4[repetDB.  AAACTGAGGACGTTATATTGTTCGGGGGTGCTATTAATAGATTTAAAATCATTAGAAGGCATGTG

                                    2080      2090      2100      2110      2120      2130
AT4G16920.2[arabidopsis_thalia  TGCTCACGTATGGAAGGCACTCAGGGCATCGTTTATTTCCCTAGTAAACTCAGATTGCTATTGTG
AT4G16950.1[arabidopsis_thalia  TGCTCACGTGTGGAAGGCACTCAGGGCATCGTTTATTTCCCTAGTAAACTCAGATTGCTATTGTG
TEdenovo-B-R2288-Map4[repetDB.  TGCTCACGTATGGAAGGCACTCAGGGCATCGTTTATTTCCCTAGTAAACTCAGATTGCTATTGTG

                                    2170      2180      2190      2200      2210      2220
AT4G16920.2[arabidopsis_thalia  CATTCTAATTTTAAGGTTGAGTATCTGGTTAAACTCAGAATGGAGAATAGTGACCTTGAGAAGCT
AT4G16950.1[arabidopsis_thalia  CATTCTAATTTTAAGGTTGAGTATCTGGTTAAACTCAGAATGGAGAATAGTGACCTTGAGAAGCT
TEdenovo-B-R2288-Map4[repetDB.  CATTCTAATTTTAAGGTTGAGTATCTGGTTAAACTCAGAATGGAGAATAGTGACCTTGAGAAGCT

                                    2260      2270      2280      2290      2300      2310
AT4G16920.2[arabidopsis_thalia  AGACTCAAGCAGATGTTTCTGCGTGGTTCCAAATATTTGAAAGAGATTCCAGATCTTTCTTTAGC
AT4G16950.1[arabidopsis_thalia  AGACTCAAGCAGATGTTTCTGCGTGGTTCCAAATATTTGAAAGAGATTCCAGATCTTTCTTTAGC
TEdenovo-B-R2288-Map4[repetDB.  AGACTCAAGCAGATGTTTCTGCGTGGTTCCAAATATTTGAAAGAGATTCCAGATCTTTCTTTAGC

                                    2350      2360      2370      2380      2390      2400
AT4G16920.2[arabidopsis_thalia  TGTAAATGCGAATCCTTGGTGACATTTCCTTCCTCGATGCAGAATGCCATTAAACTTATCTATTT
AT4G16950.1[arabidopsis_thalia  TGTAAATGCGAATCCTTGGTGACATTTCCTTCCTCGATGCAGAATGCCATTAAACTTATCTATTT
```
