# Supplementary material for: K‐mer counting and curated libraries drive efficient annotation of repeats in plant genomes
Source: Plant Genome. 2021 Sep 25;14(3):e20143. doi: 10.1002/tpg2.20143 (PMC7614178; doi:10.1002/tpg2.20143)
Supplement: Supplementary file 2 [file TPG2-14-e20143-s002.docx]

**Supplementary Tables**

**Table S1.** Contribution of individual datasets to the nrTEplants library of repeats (v0.3).

| **Dataset** | **# of sequences** |
| --- | --- |
| SunflowerTE | 58071 |
| REdat | 39509 |
| repetDB | 26006 |
| SoyBaseTE | 21766 |
| TAIR10TE | 21056 |
| EDTArice | 1844 |
| MelonTE | 1171 |
| EDTAmaize | 805 |
| TREP | 789 |
| SINEbase | 44 |
| SUNREP | 43 |

**Table S2.** Summary of classified repeats in the nrTEplants library.

| **Superfamily/family** | **count** |
| --- | --- |
| LTR/Gypsy | 45394 |
| LTR/Copia | 26009 |
| LTR | 24532 |
| TIR | 18590 |
| RC/Helitron | 9080 |
| TRIM | 6573 |
| Helitron | 4384 |
| MobileElement | 4020 |
| DNA/MuDR | 3900 |
| TIR/Tc1-Mariner | 3404 |
| LINE | 3342 |
| Unclassified | 3061 |
| TIR/Mutator | 2287 |
| DNA | 1810 |
| TIR/PIF-Harbinge | 1367 |
| DNA/En-Spm | 1088 |
| LINE/L1 | 1076 |
| Mutator | 820 |
| SINE | 785 |
| DNA/HAT | 751 |
| DIRS | 707 |
| MITE | 663 |
| rRNA | 565 |
| DNA/Harbinger | 516 |
| Other | 416 |
| DNA/Mite | 397 |
| Retroelement | 389 |
| DNA/hAT | 300 |
| TIR/hAT | 299 |
| LARD | 299 |
| DNAnona/Helitron | 260 |
| DNAnona/MULE | 251 |
| DNA/Pogo | 245 |
| DNA/TcMar | 233 |
| DNAnona/hAT | 202 |
| Other/Simple | 184 |
| TIR/PIF-Harbinger | 176 |
| nonLTR | 172 |
| DNA/Stowaway | 161 |
| RathE1_cons | 156 |
| Satellite | 155 |
| non | 130 |
| hAT | 129 |
| DNA/Mariner | 126 |
| DNA/Mutator | 122 |
| MITE/Tourist | 112 |
| TIR/CACTA | 105 |
| DNAnona/MULEtir | 92 |
| RathE3_cons | 87 |
| DNA/Tc1 | 86 |
| TIR/Mariner | 81 |
| DNAnona/CACTA | 74 |
| DNAauto/MULE | 72 |
| LINE? | 68 |
| TIR/Harbinger | 62 |
| RathE2_cons | 50 |
| Helitron/Helitron | 45 |
| non-LTR(SINE) | 43 |
| DNA/CACTA | 37 |
| DNA/Tc1-Mariner | 35 |
| MITE/Stow | 35 |
| DNA/Tourist | 32 |
| non-LTR(SINE)/I | 32 |
| Maverick | 31 |
| DNAnona/Tourist | 29 |
| LTR/TRIM | 26 |
| DNAnona/PILE | 26 |
| TIR/EnSpm/CACTA | 25 |
| LARD\|TRIM | 24 |
| DNAauto/hAT | 22 |
| DNAauto/Helitron | 17 |
| DNAauto/CACTA | 17 |
| LINE/Ukn | 15 |
| DNAauto/MLE | 14 |
| DNAnona/POLE | 14 |
| DNAauto/POLE | 12 |
| SINE\|TRIM | 10 |
| DNAnona/CACTG | 10f |
| non-LTR(SINE)/Jokey | 10 |
| LTR\|TIR | 10 |
| DNAnona/MLE | 8 |
| DIRS\|TIR | 8 |
| DNAauto/PILE | 8 |
| non-LTR(SINE)/Pan | 8 |
| DNA/hAT-Ac | 8 |
| TIR/PONG | 8 |
| non-LTR(SINE)/L1 | 8 |
| Helitron\|LARD | 7 |
| LTR/Echo | 5 |
| PLE | 5 |
| LTR/Halcyon | 5 |
| Other/Centromeric | 4 |
| Helitron\|TRIM | 3 |
| LTR/Solo | 3 |
| DNA/Helitron | 3 |
| LTR\|DIRS | 3 |
| DNAnona | 3 |
| Crypton | 2 |
| SINE\|LARD | 2 |
| Evirus/ERTBV | 2 |
| TIR/PiggyBac | 2 |
| TIR\|Maverick | 2 |
| DNAauto/CACTG | 2 |
| subtelomere/4-12-1 | 1 |
| Satellite/rice | 1 |
| TIR/P | 1 |
| Evirus/ERTBV-A | 1 |
| Centro/tandem | 1 |
| non-LTR(SINE)/R2 | 1 |
| DNAtransposon | 1 |
| non-LTR(SINE)/Chronos | 1 |
| knob/TR-1 | 1 |

**Table S3.** Wheat cultivars from [(Walkowiak et al., 2020)](https://sciwheel.com/work/citation?ids=10085575&pre=&suf=&sa=0) annotated with Red [(Girgis, 2015)](https://sciwheel.com/work/citation?ids=3711102&pre=&suf=&sa=0).

| **Cultivar** | **Assembled**  **genome size (Mbp)** | **% repeated content** |
| --- | --- | --- |
| Arinalrfor | 10.17 | 81.24 |
| Jagger | 10.16 | 81.57 |
| Julius | 10.16 | 82.45 |
| Lancer | 10.16 | 81.16 |
| Landmark | 10.16 | 82.04 |
| Mace | 10.16 | 81.41 |
| Mattis | 10.17 | 78.56 |
| Norin61 | 10.15 | 83.11 |
| Stanley | 10.15 | 83.92 |

**Table S4.** Intersection percentages among repeats called by Red [(Girgis, 2015)](https://sciwheel.com/work/citation?ids=3711102&pre=&suf=&sa=0) and repeated sequences called by RepeatMasker (Smit et al., 2015) with nrTEplants, REdat (Nussbaumer et al., 2013) and custom RepMod libraries [(Smit et al., 2015)](https://sciwheel.com/work/citation?ids=9955253&pre=&suf=&sa=0), dustmasker [(Morgulis et al., 2006)](https://sciwheel.com/work/citation?ids=1336232&pre=&suf=&sa=0) and trf [(Benson, 1999)](https://sciwheel.com/work/citation?ids=966900&pre=&suf=&sa=0).

|  | **REdat** | **nrTEplants** | **dust** | **trf** | **RepMod** |
| --- | --- | --- | --- | --- | --- |
| *Arabidopsis thaliana* | 26.1 | 46.9 | 11.9 | 6.6 | 33.4 |
| *Arabidopsis halleri* | 34.9 | 56.2 | 13.2 | 5.9 |  |
| *Prunus dulcis* | 12.8 | 81.9 | 9 | 9.4 | 87.3 |
| *Brachypodium distachyon* | 75.8 | 74.7 | 3.1 | 4.4 | 88.8 |
| *Brassica rapa* | 13 | 57.4 | 19.6 | 9.5 | 67.8 |
| *Trifolium pratense* | 23 | 18.5 | 15.6 | 6.5 |  |
| *Arabis alpina* | 30.1 | 87.5 | 6.6 | 6.8 |  |
| *Cucumis melo* | 15.1 | 85.7 | 11.4 | 10.7 | 94.3 |
| *Citrullus lanatus* | 11.9 | 30.5 | 9.2 | 6.6 |  |
| *Oryza sativa* | 74.1 | 83.8 | 7.3 | 7.9 |  |
| *Setaria viridis* | 38.7 | 40.6 | 3.3 | 5.2 |  |
| *Vitis vinifera* | 15.9 | 82.5 | 14.6 | 10.2 | 90.2 |
| *Rosa chinensis* | 14.2 | 35.9 | 4.7 | 6 | 90 |
| *Camelina sativa* | 31.3 | 43.7 | 8.8 | 7.3 | 71.8 |
| *Malus domestica* | 17.4 | 87.6 | 5.4 | 9.1 |  |
| *Olea europaea* | 33.9 | 35.8 | 4.9 | 38.2 |  |
| *Zea mays* | 73.4 | 77.1 | 1.9 | 4.1 | 96.2 |
| *Helianthus annuus* | 12.9 | 32.8 | 1.6 | 2.1 |  |
| *Aegilops tauschii* | 82 | 83.1 | 2.2 | 4.7 | 96.8 |
| *Triticum turgidum* | 84.7 | 85.4 | 2 | 3.3 |  |

**Table S5.** Median number of repeat k-mers with 20+ copies overlapping 500bp up/downstream regions in plant genomes. Values were computed from twenty genomes from release 49 (November 2020) of Ensembl Plants annotated with Red [(Girgis, 2015)](https://sciwheel.com/work/citation?ids=3711102&pre=&suf=&sa=0) or RepeatMasker [(Smit et al., 2015)](https://sciwheel.com/work/citation?ids=9955253&pre=&suf=&sa=0) with libraries REdat [(Nussbaumer et al., 2013)](https://sciwheel.com/work/citation?ids=952315&pre=&suf=&sa=0) and nrTEplants. Total k-mers are also shown.

| K | **REdat (n>20)** | **nrTEplants**  **(n>20)** | **Red**  **(n>20)** | **REdat**  **(total)** | **nrTEplants**  **(total)** | **Red**  **(total)** |
| --- | --- | --- | --- | --- | --- | --- |
| 16 | 2101 | 3806.5 | 14730 | 1212499.5 | 3230821 | 6125298 |
| 21 | 1307.5 | 2285.5 | 8057 | 1178632.5 | 3150425.5 | 5925111.5 |
| 31 | 606.5 | 984 | 4256.5 | 1112352.5 | 2993877.5 | 5533979 |

**Table S6.** Enriched Pfam domains of protein-coding genes overlapping repeats called with Red [(Girgis, 2015)](https://sciwheel.com/work/citation?ids=3711102&pre=&suf=&sa=0) and RepeatMasker [(Smit et al., 2015)](https://sciwheel.com/work/citation?ids=9955253&pre=&suf=&sa=0) with libraries nrTEplants and REdat [(Nussbaumer et al., 2013)](https://sciwheel.com/work/citation?ids=952315&pre=&suf=&sa=0). Results for 10 genomes masked with RepMod custom libraries are also shown (Flynn et al., 2020). Only domains found enriched in at least three species are shown. Domains in bold are shared by all repeat-calling strategies except RepMod and correspond to Integrase core domains (PF00665), NB-ARC (PF00931), Reverse transcriptase-like (PF13456) and TIR (PF01582). Underlined domains are enriched in species annotated with RepeatMasker regardless of the library and correspond to protein kinase domains (PF07714, PF00069).

| **REdat** | | **nrTEplants** | | **Red** | | **RepMod** | |
| --- | --- | --- | --- | --- | --- | --- | --- |
| Pfam domain | count | Pfam domain | count | Pfam domain | count | Pfam domain | count |
| PF00069 | 13 | **PF00931** | 11 | **PF13456** | 3 | PF07714 | 3 |
| PF07714 | 10 | PF07727 | 6 | **PF01582** | 3 | PF00069 | 3 |
| PF00005 | 10 | PF07714 | 6 | **PF00931** | 3 |  |  |
| PF01095 | 9 | **PF01582** | 6 | **PF00665** | 3 |  |  |
| PF13947 | 8 | PF00078 | 6 |  |  |  |  |
| PF07727 | 8 | PF00069 | 6 |  |  |  |  |
| **PF00931** | 8 | PF18052 | 5 |  |  |  |  |
| PF13855 | 7 | PF14223 | 5 |  |  |  |  |
| PF01453 | 7 | PF07725 | 5 |  |  |  |  |
| PF00847 | 7 | **PF00665** | 5 |  |  |  |  |
| **PF00665** | 7 | PF17921 | 4 |  |  |  |  |
| PF00078 | 7 | PF13976 | 4 |  |  |  |  |
| PF14510 | 6 | PF13855 | 4 |  |  |  |  |
| PF14432 | 6 | PF03732 | 4 |  |  |  |  |
| PF14223 | 6 | PF00560 | 4 |  |  |  |  |
| PF08370 | 6 | PF14111 | 3 |  |  |  |  |
| PF08276 | 6 | PF13947 | 3 |  |  |  |  |
| **PF01582** | 6 | **PF13456** | 3 |  |  |  |  |
| PF00954 | 6 | PF08276 | 3 |  |  |  |  |
| PF00067 | 6 | PF08263 | 3 |  |  |  |  |
| PF17921 | 5 | PF01453 | 3 |  |  |  |  |
| PF17919 | 5 | PF00954 | 3 |  |  |  |  |
| PF17917 | 5 | PF00122 | 3 |  |  |  |  |
| PF13976 | 5 | PF00005 | 3 |  |  |  |  |
| PF11721 | 5 |  |  |  |  |  |  |
| PF08387 | 5 |  |  |  |  |  |  |
| PF08284 | 5 |  |  |  |  |  |  |
| PF08263 | 5 |  |  |  |  |  |  |
| PF07725 | 5 |  |  |  |  |  |  |
| PF07723 | 5 |  |  |  |  |  |  |
| PF03732 | 5 |  |  |  |  |  |  |
| PF00560 | 5 |  |  |  |  |  |  |
| PF00082 | 5 |  |  |  |  |  |  |
| PF19055 | 4 |  |  |  |  |  |  |
| PF17766 | 4 |  |  |  |  |  |  |
| PF08246 | 4 |  |  |  |  |  |  |
| PF02365 | 4 |  |  |  |  |  |  |
| PF01061 | 4 |  |  |  |  |  |  |
| PF00664 | 4 |  |  |  |  |  |  |
| PF00201 | 4 |  |  |  |  |  |  |
| PF00112 | 4 |  |  |  |  |  |  |
| PF00098 | 4 |  |  |  |  |  |  |
| PF00012 | 4 |  |  |  |  |  |  |
| PF14380 | 3 |  |  |  |  |  |  |
| **PF13456** | 3 |  |  |  |  |  |  |
| PF12819 | 3 |  |  |  |  |  |  |
| PF08372 | 3 |  |  |  |  |  |  |
| PF08031 | 3 |  |  |  |  |  |  |
| PF05922 | 3 |  |  |  |  |  |  |
| PF00450 | 3 |  |  |  |  |  |  |
| PF00305 | 3 |  |  |  |  |  |  |
| PF00249 | 3 |  |  |  |  |  |  |
| PF00223 | 3 |  |  |  |  |  |  |

**Table S7.** Odd ratios of NLR masking for genes overlapping > 50bp of masked sequences. Values were computed from twenty genomes from release 49 (November 2020) of Ensembl Plants annotated with Red [(Girgis, 2015)](https://sciwheel.com/work/citation?ids=3711102&pre=&suf=&sa=0) or RepeatMasker [(Smit et al., 2015)](https://sciwheel.com/work/citation?ids=9955253&pre=&suf=&sa=0) with libraries REdat [(Nussbaumer et al., 2013)](https://sciwheel.com/work/citation?ids=952315&pre=&suf=&sa=0) and nrTEplants. Results for 10 genomes masked with RepMod custom libraries are also shown (Flynn et al., 2020).

| **Species** | **NLR genes** | **NLR space (bp)** | **REdat** | **nrTEplants** | **Red** | **RepMod** |
| --- | --- | --- | --- | --- | --- | --- |
| *Arabidopsis thaliana* | 69 | 201660 | 0.77 | 2.6 | 0.71 | 2.01 |
| *Arabidopsis halleri* | 209 | 635687 | 0.56 | 2.41 | 0.62 |  |
| *Prunus dulcis* | 387 | 1354547 | 2.52 | 2.05 | 0.97 | 1.29 |
| *Brachypodium distachyon* | 344 | 1249602 | 0.46 | 0.59 | 0.46 | 0.56 |
| *Brassica rapa* | 219 | 729119 | 0.89 | 2.22 | 0.66 | 1.83 |
| *Trifolium pratense* | 553 | 1781566 | 2.65 | 4.11 | 1.06 |  |
| *Arabis alpina* | 364 | 1201405 | 0.51 | 1.46 | 0.44 |  |
| *Cucumis melo* | 89 | 294239 | 1.59 | 1.46 | 0.37 | 0.61 |
| *Citrullus lanatus* | 43 | 164993 | 1.33 | 2.44 | 0.22 |  |
| *Oryza sativa* | 45 | 169051 | 0.22 | 0.44 | 0.22 |  |
| *Setaria viridis* | 453 | 1682607 | 0.35 | 0.47 | 0.56 |  |
| *Vitis vinifera* | 739 | 2723777 | 1.54 | 1.36 | 0.98 | 1.3 |
| *Rosa chinensis* | 963 | 3347977 | 1.42 | 2.97 | 0.8 | 1.2 |
| *Camelina sativa* | 573 | 1808571 | 0.39 | 2.3 | 0.53 | 1.31 |
| *Malus domestica* | 637 | 2535932 | 1.5 | 1.63 | 0.89 |  |
| *Olea europaea* | 402 | 1095646 | 0.42 | 0.73 | 0.21 |  |
| *Zea mays* | 158 | 487237 | 0 | 0 | 0.43 |  |
| *Helianthus annuus* | 604 | 2055877 | 0.29 | 0.88 | 0.59 |  |
| *Aegilops tauschii* | 916 | 3144217 | 0 | 0.14 | 0.14 | 0.14 |
| *Triticum turgidum* | 2459 | 8351371 | 0 | 0.13 | 0.13 |  |

**Table S8.** Repeat families discovered by RepeatModeller (Flynn et al., 2020) after 7 days on a computer cluster with 20 parallel processes on twenty genomes from release 49 (November 2020) of Ensembl Plants. UF stands for unfinished after 7 days. The last column is the CPU time taken by RepeatMasker (RM) to map these repeats against the genome.

| **Species** | **Repeat families** | **Unclassified** | **RepMod CPU time (h)** | **RM CPU**  **time (min)** |
| --- | --- | --- | --- | --- |
| *Arabidopsis thaliana* | 656 | 415 | 83:22:10 | 59.9 |
| *Arabidopsis halleri* |  |  | UF |  |
| *Prunus dulcis* | 1833 | 1428 | 33:38:23 | 191.5 |
| *Brachypodium distachyon* | 1591 | 1218 | 86:48:42 | 179 |
| *Brassica rapa* | 1893 | 1504 | 132:00:54 | 234 |
| *Trifolium pratense* |  |  | UF |  |
| *Arabis alpina* |  |  | UF |  |
| *Cucumis melo* | 2325 | 2325 | 127:14:47 | 537.3 |
| *Citrullus lanatus* |  |  | UF |  |
| *Oryza sativa* |  |  | UF |  |
| *Setaria viridis* |  |  | UF |  |
| *Vitis vinifera* | 2218 | 1603 | 56:57:18 | 684.6 |
| *Rosa chinensis* | 2790 | 2218 | 61:38:48 | 638.3 |
| *Camelina sativa* | 2442 | 1730 | 144:57:56 | 723.6 |
| *Malus domestica* |  |  | UF |  |
| *Olea europaea* |  |  | UF |  |
| *Zea mays* | 1575 | 1149 | 52:32:01 | 3270.1 |
| *Helianthus annuus* |  |  | UF |  |
| *Aegilops tauschii* | 1793 | 1405 | 123:07:26 | 5546.8 |
| *Triticum turgidum* |  |  | UF |  |
