# Supplementary material for: K-mer counting and curated libraries drive efficient annotation of repeats in plant genomes
Source: Plant Genome. Author manuscript; Available in PMC 2023 Feb 14. (PMC7614178; doi:10.1002/tpg2.20143)
Supplement: Supplemental Figure 1 [file EMS164607-supplement-Supplemental_Figure_1.pdf]

```
AT4G16920.2[arabidopsis_thalia   AAAGATGAGTGGGTGAAGATGATGCCTAGGCTTCGAAATGACTCAGATGATAAAATTGAGGAAAC
AT4G16950.1[arabidopsis_thalia   AAAAAAGAGTGGATGGAGATGATGCCTAGGCTCCGAAATGTTTCGGA-GATATTA-TGA--AAAC
TEdenovo-B-R2288-Map4[repetDB.   -----------------------------------------------------------------

                                        1270      1280      1290      1300      1310      1320
AT4G16920.2[arabidopsis_thalia   AATAAAAAAAATAGAGAGTTATTTAAGTGCATTGCATGTTTCTTCAATGGTTTTAAAGTCAGTAA
AT4G16950.1[arabidopsis_thalia   CATCAAAAAGAT-CA-AGATATTTCTTTGCATTGCGTGTTTATTCAATGGTTTTGAGGTCAGTTA
TEdenovo-B-R2288-Map4[repetDB.   -----------------------------------------------------------------

                                        1360      1370      1380      1390      1400      1410
AT4G16920.2[arabidopsis_thalia   GTTGGGCTTACAATGTTGGTTGAGAAGTCCCTCATACGTATTACACCGGATGGAGATATAGAGAT
AT4G16950.1[arabidopsis_thalia   GTTGGGCTTACAATGTTGTCTGAGAAGTCCCTCATACGTATTACACCGGATGGACATATAGAGAT
TEdenovo-B-R2288-Map4[repetDB.   -----------------------------------------------------------------

                                        1450      1460      1470      1480      1490      1500
AT4G16920.2[arabidopsis_thalia   AGAGAAATTGATCGTGCAAAGTCCAAGGGTAATCCTGGAAAACGTCAATTTCTGACAAATTTTGA
AT4G16950.1[arabidopsis_thalia   AGAGAAATTGATCGTGCAAAGTCCAAGGGTAATCCTGGAAAACGTCAATTTCTGACGAATTTTGA
TEdenovo-B-R2288-Map4[repetDB.   -----------------------------------------------------------------

                                        1540      1550      1560      1570      1580      1590
AT4G16920.2[arabidopsis_thalia   AAAACTGGGACAGAAATTCTTCTTGGAATACGTTTGCCACACCCGGGATATCTTACGACAAGGTC
AT4G16950.1[arabidopsis_thalia   AAAACTGGGACAGAAACTCTTCTTGGAATACGTTTGCCATTCGAGGAATATTTTTCGACAAGGCC
TEdenovo-B-R2288-Map4[repetDB.   -----------------------------------------------------------------

                                        1630      1640      1650      1660      1670      1680
AT4G16920.2[arabidopsis_thalia   AAAGGCATGCGTAATCTCCAATATCTAGAAATTGGTTATTGGTCAGATGGGGATCTACCTCAGAG
AT4G16950.1[arabidopsis_thalia   AAAGGCATGCGTAATCTCCAATATCTAAAAATTGGTGATTGGTCAGATGGGGGTCAACCTCAGAG
TEdenovo-B-R2288-Map4[repetDB.   -----------------------------------------------------------------

                                        1720      1730      1740      1750      1760      1770
AT4G16920.2[arabidopsis_thalia   AGATTGCTAGAATGGGTTTATTGTCCGTTGAAGTCTTTGCCATCTACTTTTAGGGCGGAGTATCT
AT4G16950.1[arabidopsis_thalia   AGATTGCTAGACTGGGATGATTGTCCATTGAAGTCTTTGCCATCTACTTTTAAGGCGGAATATCT
TEdenovo-B-R2288-Map4[repetDB.   -----------------------------------------------------------------

                                        1810      1820      1830      1840      1850      1860
AT4G16920.2[arabidopsis_thalia   AAGCTTGAGAAACTGTGGGAAGGAACTCTGCCCCTTGGAAGTCTCAAGAAGATGAATTTGTGGTA
AT4G16950.1[arabidopsis_thalia   AAGCTTGAGAAACTGTGGGAAGGAACTCTGCCCCTTGGAAGTCTCAAGAAGATGAATTTGTTGTG
TEdenovo-B-R2288-Map4[repetDB.   -----------------------------GCCCCTTGGAAGTCTCAAGAAGATGAATTTGTGGTG

                                        1900      1910      1920      1930      1940      1950
AT4G16920.2[arabidopsis_thalia   GATCTTTCTTTAGCCATAAACCTCGAGGAATTAAATCTTTCTGAATGCGAATCTTTGGTGACACT
AT4G16950.1[arabidopsis_thalia   GATCTTTCTAATGCCAGAAACCTCGAGGAATTAGATCTTGAAGGATGCGAATCTTTGGTGACACT
TEdenovo-B-R2288-Map4[repetDB.   GATCTTTCTTTAGCCATAAACCTCGAGGAATTAGATCTTTCTGGATGCGAATCTTTGGTGACACT

                                        1990      2000      2010      2020      2030      2040
AT4G16920.2[arabidopsis_thalia   AAACTGAGGACGTTATATTGTTCGGGGGTGCTATTAATAGATTTAAAATCATTAGAAGGCATGTG
AT4G16950.1[arabidopsis_thalia   AAACTGAGGAAGTTACATTGTTCGGGGGTGATATTAATAGATTTAAAATCATTAGAAGGCATGTG
TEdenovo-B-R2288-Map4[repetDB.   AAACTGAGGACGTTATATTGTTCGGGGGTGCTATTAATAGATTTAAAATCATTAGAAGGCATGTG

                                        2080      2090      2100      2110      2120      2130
AT4G16920.2[arabidopsis_thalia   TGCTCACGTATGGAAGGCACTCAGGGCATCGTTTATTTCCCTAGTAAACTCAGATTGCTATTGTG
AT4G16950.1[arabidopsis_thalia   TGCTCACGTGTGGAAGGCACTCAGGGCATCGTTTATTTCCCTAGTAAACTCAGATTGCTATTGTG
TEdenovo-B-R2288-Map4[repetDB.   TGCTCACGTATGGAAGGCACTCAGGGCATCGTTTATTTCCCTAGTAAACTCAGATTGCTATTGTG

                                        2170      2180      2190      2200      2210      2220
AT4G16920.2[arabidopsis_thalia   CATTCTAATTTTAAGGTTGAGTATCTGGTTAAACTCAGAATGGAGAATAGTGACCTTGAGAAGCT
AT4G16950.1[arabidopsis_thalia   CATTCTAATTTTAAGGTTGAGTATCTGGTTAAACTCAGAATGGAGAATAGTGACCTTGAGAAGCT
TEdenovo-B-R2288-Map4[repetDB.   CATTCTAATTTTAAGGTTGAGTATCTGGTTAAACTCAGAATGGAGAATAGTGACCTTGAGAAGCT

                                        2260      2270      2280      2290      2300      2310
AT4G16920.2[arabidopsis_thalia   AGACTCAAGCAGATGTTTCTGCGTGGTTCCAAATATTTGAAAGAGATTCCAGATCTTTCTTTAGC
AT4G16950.1[arabidopsis_thalia   AGACTCAAGCAGATGTTTCTGCGTGGTTCCAAATATTTGAAAGAGATTCCAGATCTTTCTTTAGC
TEdenovo-B-R2288-Map4[repetDB.   AGACTCAAGCAGATGTTTCTGCGTGGTTCCAAATATTTGAAAGAGATTCCAGATCTTTCTTTAGC

                                        2350      2360      2370      2380      2390      2400
AT4G16920.2[arabidopsis_thalia   TGTAAATGCGAATCCTTGGTGACATTTCCTTCCTCGATGCAGAATGCCATTAAACTTATCTATTT
AT4G16950.1[arabidopsis_thalia   TGTAAATGCGAATCCTTGGTGACATTTCCTTCCTCGATGCAGAATGCCATTAAACTTATCTATTT
```
